# Co-Encapsulated Synbiotics and Immobilized Probiotics in Human Health and Gut Microbiota Modulation

**DOI:** 10.3390/foods10061297

**Published:** 2021-06-04

**Authors:** Monika Kvakova, Izabela Bertkova, Jana Stofilova, Tor C. Savidge

**Affiliations:** 1 Department of Experimental Medicine, Faculty of Medicine, P.J. Safarik University in Kosice, Trieda SNP 1, 04011 Kosice, Slovakia; izabela.bertkova@upjs.sk (I.B.); jana.stofilova@upjs.sk (J.S.); 2 Department of Biochemistry, Faculty of Science, P.J. Safarik University in Kosice, Moyzesova 11, 04001 Kosice, Slovakia; 3 Department of Pathology and Immunology, Baylor College of Medicine, Houston, TX 77030, USA; tor.savidge@bcm.edu; 4 Texas Children’s Microbiome Center, Department of Pathology, Texas Children’s Hospital, Houston, TX 77030, USA

**Keywords:** co-encapsulated synbiotics, immobilized probiotics, prebiotics, synergism, gut microbiota modulation, health benefits

## Abstract

Growing interest in the development of innovative functional products as ideal carriers for synbiotics, e.g., nutrient bars, yogurt, chocolate, juice, ice cream, and cheese, to ensure the daily intake of probiotics and prebiotics, which are needed to maintain a healthy gut microbiota and overall well-being, is undeniable and inevitable. This review focuses on the modern approaches that are currently being developed to modulate the gut microbiota, with an emphasis on the health benefits mediated by co-encapsulated synbiotics and immobilized probiotics. The impact of processing, storage, and simulated gastrointestinal conditions on the viability and bioactivity of probiotics together with prebiotics such as omega-3 polyunsaturated fatty acids, phytochemicals, and dietary fibers using various delivery systems are considered. Despite the proven biological properties of synbiotics, research in this area needs to be focused on the proper selection of probiotic strains, their prebiotic counterparts, and delivery systems to avoid suppression of their synergistic or complementary effect on human health. Future directions should lead to the development of functional food products containing stable synbiotics tailored for different age groups or specifically designed to fulfill the needs of adjuvant therapy.

## 1. Introduction

The host’s microbiota is a complex ecosystem of bacteria, eukaryotic microbes, viruses, and archaea coexisting within the body and also on tissue surfaces. In these locations, the microbiota plays important roles in a variety of physiological activities, including digestion, metabolism, immune reactions, biosynthesis of numerous compounds, elimination of toxins, regulation of the gut-brain axis function, and even disease pathogenesis. The majority of these microbial communities reside within the gut and are influenced by the mode of birth, infant feeding, genetic background, and lifestyle, including diet, exercise, stress, medication, and overall health of the host. Generally, the sum of the unique microbial genes in the gut is called the gut microbiome [1,2,3,4]. The majority of symbiotic bacteria that colonize the human gut can be classified into several phyla, comprising Bacteroidetes and Firmicutes, followed by Proteobacteria, Actinobacteria, Fusobacteria, Verrucomicrobia, and Spirochaetes [2,5]. Gut microbial populations can vary significantly between individuals, even in healthy subjects. However, there is little doubt that basic physiological functions need to be maintained in the case of disruption of microbial composition, and this is achieved through a set of core microorganisms [5,6]. Unfavorable alterations in microbial composition and function are characteristics of many disease states and are known as dysbiosis. Although there is growing evidence that dysbiosis is associated with various human diseases, such as inflammatory bowel diseases, irritable bowel syndrome, allergies, asthma, metabolic syndrome, diabetes, obesity, cardiovascular diseases, cancer, and depression or anxiety, it is not well known if and how this contributes to pathogenesis [7,8,9,10,11,12,13]. Based on the increasing number of diverse disorders that are associated with dysbiosis, including antibiotic resistance, there is great interest in identifying ways to modulate the gut microbiota in order to find a preventive strategy for sustaining gut health. Well-known approaches to naturally modulate gut microbiota composition include a balanced diet that is rich in fresh fruits and vegetables, grains, and fermented products. Recent studies also suggest that regular exercise can alter gut microbial communities [14,15,16]. Modern approaches, such as the administration of probiotics and prebiotics—alone or mixed as synbiotics—in sufficient concentrations, as well as fecal microbiota transplantation (FMT) intervention in severe cases, have been intensively studied and may have health benefits, although protective mechanisms are not clearly understood [17,18,19]. Currently, there is growing interest in functional innovative products as ideal carriers, e.g., nutrient bars, yogurt, chocolate, juice, ice cream, cheese, or even sausage, for co-encapsulated synbiotics, to ensure the daily intake of probiotics and prebiotics needed to maintain a healthy microbiota and good mental health as well as boost immunity [20,21,22,23,24,25]. In connection with the COVID-19 outbreak, Zuo et al. [26] showed that the gut microbiome is perturbed after SARS-CoV-2 infection and outlined the existence of the gut-lung axis, in which the gut microbiota is metabolically able to affect lung function [27]. This possible link between SARS-Co-V-2 infection and gut microbiome or even lung microbiome alterations was reviewed elsewhere [28,29,30,31,32]. However, further research is needed to confirm whether probiotics or synbiotics positively interfere with COVID-19 disease severity or possess antiviral efficacy [33,34]. Further multi-center studies are also needed to establish that COVID-19-associated dysbiosis is not caused by medications used to treat the underlying illness.

**Figure 1:**
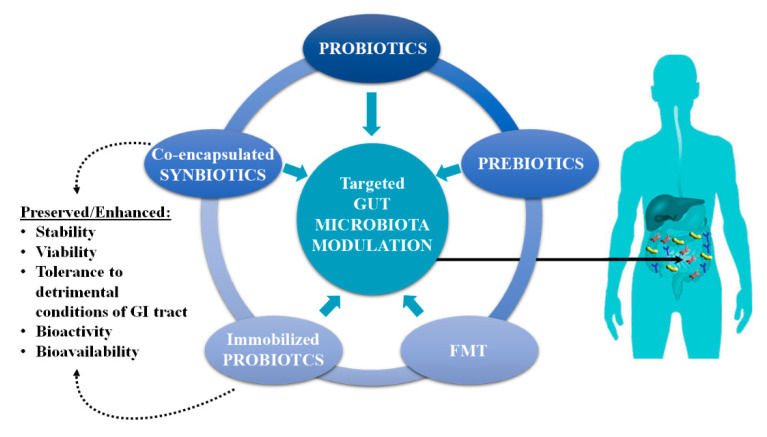
Modern approaches used in the targeted gut microbiota modulation and benefits of co-encapsulated synbiotics and immobilized probiotics.

This review focuses on modern approaches that are currently being developed to modulate the gut microbiota, with an emphasis on the health benefits mediated by co-encapsulated synbiotics and immobilized probiotics (Figure 1). The impact of processing, storage, and simulated gastrointestinal conditions (sGIC) on the viability and bioactivity of probiotics, with or without prebiotics such as omega-3 polyunsaturated fatty acids (PUFAs), phytochemicals, and dietary fibers, using various delivery systems are considered.

## 2. Gut Microbiota Modulation

### 2.1. Modulation by Probiotics

Probiotics are proposed as alternatives to antimicrobial drugs and adjuvant therapy in combating disease associated with gut dysbiosis. Probiotics are viable, non-pathogenic microorganisms that, when present in sufficient amounts, may confer health benefits on the host [35]. Various formulations, including capsules, tablets, powders, and food products containing probiotics, are commercially available today. Efficient delivery of probiotics to the intestine is crucial in achieving therapeutic efficiency because of the low bioavailability associated with the oral delivery of probiotics. To improve the health of the host through the beneficial action of bacterial species, it is widely accepted that the number of viable probiotic cells present in any type of formulation must attain a concentration equal to or greater than 10^6^–10^7^ CFU per gram or mL [36,37,38]. *Lactobacillus* and *Bifidobacterium* genera are the most frequently used bacteria for probiotic purposes, but other lactic-acid-producing bacteria, including *Enterococcus*, *Streptococcus*, and *Lactococcus,* are also widely used. In addition, next generation probiotic candidates, such as *Akkermansia muciniphila*, some *Bacillus* spp. and *Propionibacterium freudenreichii*—which belong to GRAS (Generally Recognized As Safe) microorganisms—and yeasts of the genus *Saccharomyces* exhibit probiotic characteristics [39,40,41]. For a strain to be considered probiotic, it needs to be resistant to host-induced stressors, where it should show an ability to adhere and/or proliferate at the site of action. It should also be safe to use and be deficient in any transferable antimicrobial resistant traits, though it may exhibit antimicrobial activity [42,43]. The beneficial effects of probiotics include sustaining a healthy microbiome, preventing pathogenic infections, and restoring microbial dysbiosis. Additional beneficial effects on the host are also favorable probiotic traits, including stabilizing and enhancing intestinal barrier function and producing anti-mutagenic, anti-carcinogenic, and other biologically important compounds such as short-chain fatty acids (SCFA), B-group vitamins, or vitamin K [44,45]. Moreover, probiotics are able to sense and regulate the action of secondary metabolites (e.g., bacteriocins, enzymes, and exopolysaccharides). Probiotic rich diets, adjunctive probiotic supplementation, and the prescription of personalized probiotics based on previous microbial analysis (targeted gut microbiota modulation) are linked with the prevention and potential treatment of several severe disorders, such as inflammatory bowel diseases, colorectal cancer, obesity, diabetes, and cardiovascular diseases as well as food allergies, depression, and brain function [17,46,47,48,49,50,51,52,53,54]. As the worldwide incidence of food allergies is increasing, there is an urgent need for well-controlled studies that demonstrate the positive outcomes of probiotics. For example, Tan-Lim et al. [55] determined the effectiveness of probiotics in a food allergy treatment for children. *Lactobacillus rhamnosus GG* administration likely helped infants to tolerate cow’s milk. Ma et al. [52] studied the protective effects of a lyophilized probiotic mixture (*L. paracasei, L. reuteri, L. gasseri, L. salivarius, L. johnsonii, Bifidobacterium animalis*) against food allergies. Ovalbumin-induced allergic responses were suppressed after treatment with probiotics, and this provided molecular insight into the probiotic mechanism of action. However, to ensure the long-term viability and efficacy of probiotics during processing, storage, and delivery to the site of action within the human body, advanced technologies such as microencapsulation or immobilization are recommended and have been extensively studied in the past decades [56,57,58,59].

### 2.2. Modulation by Prebiotics

The International Scientific Association for Probiotics and Prebiotics (ISAPP) offers expertise in microbiology, nutrition, and clinical research and recently updated the definition of prebiotics to “a substrate that is selectively utilized by host microorganisms conferring a health benefit”. This definition was expanded from the previous prebiotic definition of carbohydrate-based substances to non-carbohydrate ones, such as PUFAs, polyphenols, etc. [60]. Prebiotics naturally exist in diverse vegetables, fruits, and other sources, including asparagus, sugar beet, garlic, chicory, onion, Jerusalem artichoke, banana, honey, blueberry, barley, wheat, tomato, potato, rye, soybean, peas, and beans, and recently have been identified in tea, vegetable oils, seaweeds, and microalgae [61]. Prebiotics, a group of nutrients comprised mainly of non digestible oligosaccharides fructans and galactans and some PUFAs or polyphenols, rely on selective utilization by microorganisms, which results in shaping and modulating the host’s gut microbiota [18,62]. It was confirmed that prebiotics are utilized as selective substrates not only by groups of microorganisms present in the colon but also by microbes colonizing other body sites outside of the gastrointestinal tract (GI tract), which are associated with promoting host health [39,60]. The potential benefits related to prebiotics include shifting gut microbiota composition (e.g., the product of the fermentation process might stimulate or inhibit the growth of other microorganisms) along with the release of microbial metabolites such as SCFA [60,61,63]. Hiel et al. [64] evaluated the impact of daily consumption of vegetables rich in inulin-type fructans on the gut microbiota. An increase in the genus *Bifidobacterium* was observed, and the same effect was also demonstrated after consumption of Jerusalem artichokes processed in different forms [65,66]. Furthermore, the growth of *Bifidobacterium longum* subsp. *longum* and, to a lesser extent, *B. pseudocatenulatum*, *B*. *bifidum*, and *B*. *adolescentis* at the species level was observed. However, *Bifidobacterium* abundance returned to baseline levels three weeks after the end of the treatment [64]. Kjølbæk et al. [67] investigated the diet-induced effects of arabinoxylan-oligosaccharides and PUFAs on gut microbiota modulation. During arabinoxylan-oligosaccharides supplementation, increased abundance of the species *B. adolescentis*, *B. longum,* and members of the genera *Faecalibacterium*, *Ruminococcus*, *Dorea*, and *Eubacterium* was observed. Furthermore, an increased abundance in butyrate-producing species such as *Roseburia*, *Coprococcus,* and *Anaerostipes* and bacteria belonging to the Clostridia class, particularly *Eubacterium rectale*, *Faecalibacterium prausnitzii,* and *Eubacterium hallii,* was observed because of the cross-feeding process. Reduction in both *Rikenellaceae* and *Porphyromonadaceae* was also observed. However, fourweek PUFAs intake did not induce any significant shift in the gut microbiota composition. In the study by Vigsnæs et al. [68], *Bifidobacterium* spp. and *Lactobacillus* spp. were selectively increased, accompanied by a high production of volatile metabolite acetate, after fermentation of arabino-oligosaccharides or fructo-oligosaccharides (FOS) by fecal microbiota obtained from patients with ulcerative colitis. However, the relative abundance of the butyrate-producing species *F. prausnitzii* and the butyrate-producing bacterial groups *Clostridium coccoides* (cluster XIVa) and *Clostridium leptum* (cluster IV) was decreased after incubation with arabino-oligosaccharides as well as FOS [68]. Analyzed samples comprising potato starch showed rapid growth of *Streptococcus* and *Prevotella* during fermentation; however, in mixed samples with maize starch, these two genera decreased, whereas *Ruminobacter*, *Succinivibrio* and unclassified *Lachnospiraceae* gradually increased. The study also pointed out that structural properties of the substrate itself can shift microbiota community composition and function [69]. Combination of isomalto-oligosaccharides with green tea extract (GTE) rich in polyphenols selectively enhanced the abundance of *Lactobacillus, Bifidobacterium, Akkermansia, Parabacteriodes, Roseburia, Rikenella, Ruminococcus,* and *Sutterella,* while it decreased *Butyricimonas, Desulfovibrio, Dorea, Mucispirillum, Neisseria, Odoribacter, Prevotella, Paraprevotella,* and *Streptococcus*. It was also observed to restore the Firmicutes-to-Bacteriodetes ratio [70]. Monofloral honey from *Prunella vulgaris* is rich in a variety of polyphenolic compounds, which positively modulated the Firmicutes-to-Bacteroidetes ratio and restored *Lactobacillus* spp. populations in rats with induced colitis [71]. Polyphenols can also inhibit the growth and adhesion of pathogenic bacteria. For example, the green and black tea extracts, epigallocatechin-3-gallate (EGCG) and theaflavins, inhibit *Fusobacterium nucleatum* biofilm formation and adhesion to oral epithelial cells and matrix proteins [72]. Natural flavonoid isoorientin, with its antioxidant and anti-inflammatory properties, inhibited the growth of inflammation-induced pathogenic genera *Alistipes*, *Helicobacter*, and *Oscillibacter* [73]. More studies of different types of non-encapsulated and encapsulated polyphenols with an impact on gut microbiota modulation are summarized in the review article of Shi et al. [74]. Considering the safety and health benefits of prebiotics on the host microbiota, overall well-being, and long-term health, prebiotics should be consumed on a daily basis alone, mixed, or in association with probiotics, as a rational synbiotic strategy, since consumption from dietary sources is inevitable.

### 2.3. Modulation by FMT in Severe Dysbiotic States

FMT is an investigational therapy for administration of fecal microbiota from a healthy person (donor) to a patient with dysbiosis. The aim of FMT is to restore the composition and function of the patient’s microbial ecosystem to its healthy characteristics. However, healthy gut microbiota composition varies among different populations and depends on the lifestyle of an individual [19]. The European Medicines Agency (EMA) has left decision-making about the use of FMT in the hands of its member states, while in the United States, FMT is not FDA-approved, because its use has been associated with adverse outcomes in susceptible patients. Nevertheless, FMT is highly effective therapeutic alternative for *Clostridioides difficile* infection and could be a promising therapeutic approach in patients with inflammatory bowel diseases, irritable bowel syndrome, metabolic syndrome, and other dysbiotic diseases with potentially serious health consequences [75,76,77,78,79,80]. Several of these clinical trials demonstrated that FMT therapy induced positive changes in the composition of microbiome, making it more comparable to a healthy community. However, FMT is associated with the risk of transmission of pathogens, especially antibiotic-resistant strains, which pose a potential risk to recipients. Before application of FMT, a very careful selection and screening of potential donors is required to minimize the recipient health risks due to the transfer of infectious agents [81,82].

Another interesting future perspective of how to apply FMT, is to use human gut microbiota cultured anaerobically in vitro as a source of well-defined transplant material to avoid transmission of pathogens. Bioreactors such as Simulator of the Human Intestinal Microbial Ecosystem (SHIME) or mucosal SHIME (M-SHIME), Lacroix model, EnteroMix and TIM-2 dynamic computer-controlled in vitro model of the proximal colon enable a complex, well-defined, and stable microbiome community structure with good metabolic activity. However, the use of these in vitro models is facing significant limitations, such as the aforementioned stable microbiome community structure and lack of physiological host environment, e.g., stress factors, varied diet, and presence of antibodies or antimicrobial agents [83,84,85]. Furthermore, it is not clear how well these culture or bioreactor adapted communities will engraft in the host, and further studies that promote transfer and engraftment are needed.

## 3. Co-Encapsulated Synbiotics

### 3.1. Synbiotics

The International Scientific Association for Probiotics and Prebiotics (ISAPP) updated the definition of synbiotics to “a mixture comprising live microorganisms and substrate(s) selectively utilized by host microorganisms that confers a health benefit on the host”. Furthermore, two subsets of synbiotics were specified. A “complementary synbiotic” is a synbiotic composed of a probiotic combined with a prebiotic, where both components work independently. A “synergistic synbiotic” is a synbiotic in which the substrate is designed to be selectively utilized by the co-administered microorganism(s) [86]. Evidence to suggest the synergistic and complementary effects of probiotics together with prebiotics on a gut microbial composition is strong. These studies showed that synbiotics can adjust the Firmicutes-to-Bacteroidetes ratio, inhibit harmful bacteria by direct antagonism (such as *Klebsiella*, *Escherichia coli,* and *C. difficile*) and, by competitive exclusion, accelerate the recovery of a healthy gut microbiome, e.g., by maintaining intestinal pH, producing important metabolites, and promoting recovery of the gut mucosal barrier. It should be noted that the positive effects of probiotics and prebiotics depend on their suitable combination, which requires consideration of strain specificity and antimicrobial activity. Synbiotics can help to balance the gut microbiota by regulating specific gut microorganisms, and this opens the door for the development of new types of functional foods with higher precision impact than nutritional supplements or other products rich in synbiotics. Furthermore, synbiotics have the potential to help combat multidrug-resistant microorganisms [87,88,89,90]. Several clinical trials were held to confirm or disprove the potential health benefits of synbiotics. Neyrinck et al. [91] confirmed that synbiotics administered to middle-aged subjects significantly decreased the number of days of abdominal discomfort and proinflammatory status that naturally is associated with aging. Middle-aged subjects were randomized to take synbiotics (*Bifidobacterium animalis* subsp. *lactis* and FOS) or a placebo for 30 days. Although 16S rRNA gene sequencing of DNA extracted from stool demonstrated that synbiotic treatment had no impact on gut microbiota composition, plasma pro-inflammatory cytokines (IL-6, IL-8, IL-17a and INF-γ) were significantly reduced after 30 days of synbiotic supplementation. This observation could reflect the inadequacy of 16S rRNA sequencing of fecal specimens to accurately detect probiotic strains or reflect compositional changes in the proximal intestine. Phavichitr et al. [92] studied the influence of synbiotics (*Bifidobacterium breve* M-16V and galacto-oligosacharides (GOS)/FOS (9:1)) at doses closer to the bacterial cells present in human milk on intestinal bifidobacteria relative abundance, reduction of potential pathogens, and gut physiological conditions of infants. This synbiotic mixture successfully created an infant-type gut environment rich in *Bifidobacterium* species and reduced the number of *C. difficile*, resulting in a gut microbiota composition closer to the breast-fed reference group. Effects of synbiotic supplementation were also studied in patients with chronic kidney disease [93], nonalcoholic fatty liver disease [94], autoimmune disease [95], diarrhea [96], and metabolic syndrome [97]. One recommended approach to maintain the gut microbiome is daily consumption of functional food. Consumption of synbiotically fortified yogurt (*Streptococcus thermophilus, Lactobacillus delbrueckii* subsp. *bulgaricus*, *Bifidobacterium animalis* subsp. *lactis BB-12* enriched with whey protein, inulin, calcium, and vitamin D_3_) for ten weeks significantly reduced body fat mass and improved body composition, blood pressure, insulin sensitivity, and lipid profiles in obese patients with metabolic syndrome [98].

To further improve oral delivery of synbiotics and to secure their stability and viability as well as targeted release in the intestine involves co-encapsulation. The incorporation of probiotics and prebiotics such as omega-3 PUFAs, γ-aminobutyric acid (GABA), phytochemicals, dietary fibers, and micronutrients carried by single delivery matrix into functional foods or other products can confer health benefits via gut microbiota modulation [29,99,100].

### 3.2. Technologies and Carrier Materials Used in Fabrication of Co-Encapsulated Synbiotics

The most important consideration for ensuring that probiotics reach their target site after oral supplementation is survival following transit through the harsh acidic environment of the stomach, thereby permitting adequate colonization and proliferation [101]. It has been reported that the microencapsulation of probiotics into polymeric microcapsules successfully protects the probiotics from the harsh and changing conditions of the GI tract. Thus, microcapsules direct the delivery of living cargo without it losing its functionality to the target site [102,103]. Microencapsulation in general is a process in which not only the probiotic cells but also enzymes, natural bioactive substances, gaseous materials, etc. are incorporated into an encapsulating matrix or membrane [104,105]. Microcapsules can protect cargo from degrading factors contained within the ambient environment during the passage through the GI tract and promote its release at controlled rates under particular conditions, usually in the colon. Microcapsules also protect the cargo during the stabilization process and storage at a wide range of temperatures and can extend shelf-life considerably. In addition, microencapsulation of bioactive substances is designed to improve their low bioavailability in the host, mask their unpleasant flavor, expand the application range, and increase overall acceptability [106,107,108,109]. The biopolymer used for encapsulation should be permeable to nutrients and metabolites in order to maintain cell viability of the cargo. Biopolymer must also be non-cytotoxic, as well as non-antimicrobial to ensure that the host and its microbiota are not adversely affected [40,110,111,112]. The encapsulation efficiency and delivery of the cargo with the desired viability and bioactivity to the site of action depends on the composition and structure of the wall material and also on the proper selection of co-encapsulation technology. The desired delivery system should be able to release cargo under specific conditions, such as change of pH, enzymatic activity, ionic strength, or temperature [113,114]. The main biocompatible and food-grade carrier materials for the co-encapsulation purposes of synbiotics are alginate [115], chitosan [116], pectin [117], gelatin [118], starch [119], gum Arabic [120], whey protein [121], and lipid carriers [122] as well as various blends of these materials [42,123,124]. These encapsulation materials are also well-described in reports by Rodrigues et al. [39], Shori [101], Arslan et al. [125], and Sarao and Arora [126]. Recently, numerous studies have shown that incorporation of prebiotics like inulin, hi-maize, trehalose, resistant starch, etc. into the encapsulation wall material increases its resistance and the preserved viability of probiotics in extreme environments of the GI tract [127,128,129]. Selecting the right co-encapsulation technology is therefore important. This topic has been extensively reviewed [99,130,131,132,133,134], and so herein we only present a short review of the main techniques that are employed to co-encapsulate probiotics with bioactive substances in a single delivery format: spray drying [135], freeze drying [136], spray chilling [122], emulsification [137], extrusion [138], and coacervation [139].

#### 3.2.1. Co-Encapsulation with Omega-3 PUFAs and GABA

Consumption of prominent bioactive compounds such as omega-3 PUFAs in appropriate levels may trigger multiple health benefits, including prevention of cardiovascular disease, certain types of cancer, depression, non-alcoholic fatty liver disease, type-2 diabetes, obesity, and inflammation-mediated disorders [140,141,142,143].Omega-3 PUFAs are naturally occurring bioactive lipids, richly contained in fish products including oils (namely eicosapentaenoic acid (EPA) and docosahexaenoic acid (DHA)) and in plants and certain vegetable oils, such as flaxseed oil (alpha linolenic acid) [144,145,146]. Western-style diets do not meet the levels of omega-3 PUFAs required to fulfill the recommended daily intake for beneficial effects on human health. Therefore, incorporation of omega-3 PUFAs into various food products and their promotion as an important component of the human diet are needed [147,148,149].

The development of products comprising omega-3 PUFAs and probiotic strains together in a single microcapsule is an emerging area of research, because functional food products containing these particular bioactive ingredients separately have reported health benefits [150]. Microencapsulation of omega-3 PUFAs is one way to facilitate the incorporation of hydrophobic substances into functional food, thereby minimizing oxidative degradation, enhancing bioavailability, and allowing their use in stable and easy-to-handle formulations [114,147]. Studies combining both components show that PUFAs enhance the action of probiotics and vice versa, since probiotics can modulate the metabolism of dietary lipids. It was shown that PUFAs can affect the adhesion of lactobacilli in the gut, which is in line with studies suggesting that dietary PUFAs can affect the gut microbiota’s ability to adhere to the gut mucosa, possibly by modifying intestinal membrane fatty acid composition [151,152,153]. Indeed, PUFAs and probiotic supplements are being used as adjuvant therapy in inflammatory bowel diseases, allergies, rheumatoid arthritis, and obesity, with promising results. Both probiotics and PUFAs play an important role in modulating the intestinal immune system and are related to local and systemic inflammatory mechanisms [154,155]. In a randomized controlled trial, Kobyliak et al. [156] studied the intake efficiency of multi-probiotics enriched with omega-3 PUFAs as an adjuvant to the standard anti-diabetic therapy in individuals with type-2 diabetes. Supplementation once daily for eight weeks led to a significant reduction of insulin resistance, markers of chronic systemic inflammation, body weight, and body mass index as well as improved glycemic index profiles compared with placebo controls. Eratte et al. [139] reported that whey protein isolate–gum Arabic complex coacervates could successfully co-encapsulate tuna or coconut oil with *Lactobacillus casei* 431 and synergistically enhance the oxidative stability of omega-3-rich tuna oil. In addition, the viability and function of *L. casei* was observed in these spray-dried microcapsules during 90 days of storage. Free cells lost all viability within 1.5 h in the sGIC, but only a 1.5 log CFU/g loss of viability of probiotics was observed in co-encapsulated form. Moreover, the cell surface hydrophobicity and the ability of *L. casei* to adhere to the intestinal wall was significantly increased by co-encapsulation with omega-3 PUFAs [139,157,158]. Vaziri et al. [159] successfully co-encapsulated DHA-rich oil with *Lactobacillus plantarum* PTCC1058 by the extrusion-freeze-drying technique; the highest viability after *L. plantarum* co-encapsulation of 88.66% was seen when the carrier material was 0.39% gelatin, 0.55% pectin, and 1.06% alginate. Encapsulation efficiency of DHA-rich oil in microcapsules was 69.37%, and the survivability of *L. plantarum* under sGIC varied from 80.53 to 90.02%, depending on carrier material composition. Vega-Sagardia et al. [160] used vegetable oil to obtain information about the influence of the oil on bacterial viability. *Lactobacillus fermentum* UCO-979C counts in microcapsules with oil increased from 1.77 × 10^7^ to 1.55 × 10^9^ CFU/g. Alginate-Xantan gum-oil microcapsules containing bacteria biofilms released small quantities of probiotic bacteria when exposed to pH 3.0 for 90 min but also maintained their *H. pylori* inhibitory activity.

GABA is a naturally occurring amino acid, but it is non-proteinogenic in nature. GABA is a bioactive inhibitor of neurotransmission in the mammalian central nervous system. It is generally found in tea, vegetables, cereals, and fermented foods such as kimchi, miso, and tempeh, but only in small amounts. GABA is also sold as a dietary supplement in many countries, because it has relaxing, anti-anxiety, anti-cancer, and anti-diabetic effects, although there are doubts that it is able to cross the blood–brain barrier. On the other hand, low levels of GABA are linked to insomnia, anxiety, and weaker immune systems [161,162]. To enhance the nutritional potential of GABA, Pandey et al. [162] incorporated GABA together with probiotic *Lactobacillus plantarum* NCDC 414 in a single microcapsule composed of inulin, dextran, and maltodextrin using spray-drying. The optimal composition of microcapsules exhibited encapsulation efficiencies of 84.22% and 99.21% for GABA and *L. plantarum*, respectively. No significant differences in the viability of *L. plantarum* and GABA retention were found after 120 days of storage at 4 °C. Co-microencapsulation of these two substances has the potential for the development of a new kind of brain booster, although its impact on the peripheral nervous system function still needs to be evaluated.

#### 3.2.2. Co-Encapsulation with Phytochemicals

Phytochemicals are bioactive compounds produced by plants, with ingestion linked to a reduction in the risk of major chronic diseases, including certain types of cancer as well as cardiovascular and neurodegenerative diseases [163]. They are commonly present in fruits, vegetables, grains, nuts, and legumes. Phytochemicals are classified into various groups, including carotenoids, cannabinoids, polyphenols (include flavonoids, stilbenes, tannins, lignans, and phenolic acids), alkaloids, curcuminoids, nitrogen-containing compounds, and organosulphur compounds. Flavonoids can be subdivided into flavonols, flavones, flavan-3-ols, flavanones, isoflavones, anthocyanins, and chalcones [106,108,164].

Polyphenols are common in the human diet, as they are abundantly present in a broad range of consumed fruits and vegetables as well as in products such as tea, coffee, wine, and chocolate. Thus, polyphenols are emerging as suitable prebiotic and synbiotic agents. The biological properties and possible beneficial effects of polyphenols are dependent on their biotransformation by gut microbiota and enterocyte enzymes into more bioavailable and simple forms in order to be easily absorbed by the GI tract [19,165,166]. This gives rise to numerous valuable benefits for the consumer, including a vast array of protective effects against viruses, bacteria, and protozoan parasites. Enzymatic transformations in the GI tract include elimination of glycosidic tailoring by gut microbiota of diverse genera (*Lactobacillus, Eubacterium*, and *Bifidobacterium*), resulting in the formation of aglycones [167]. A few articles deal with the possible pathways of microbial metabolism of polyphenols, with a particular emphasis on the finally absorbed compounds and their potential impact on human health [167,168]. There is evidence from animal and human studies that certain doses of selected polyphenols may modify gut microbial composition, and while some bacterial groups can be inhibited, other microorganisms benefit and expand [169]. For example, tea phenols and their derivatives have significantly reduced the growth of known pathogens such as *C. difficile, C. perfringens,* and *Bacteroides* spp., while commensal anaerobes such as *Clostridium* spp. and *Bifidobacterium* spp. and certain probiotics such as *Lactobacillus* spp. are less affected [170]. Song et al. [170] investigated the metabolic effect of red pitaya fruit (*Hylocereus polyrhizus*) β-cyanins on high-fat diet-fed mice and detected protective effects against diet-induced obesity and its related metabolic disorders. β-cyanins are also able to modulate gut microbiota, especially decreases in the ratio of Firmicutes and Bacteroidetes, with increases in the relative abundance of *Akkermansia* spp. *Akkermansia muciniphila* is a gram-negative anaerobic mucin-degrading bacterium and is reduced in several inflammatory and metabolic disorders, including obesity, type-2 diabetes, and inflammatory bowel diseases. *A. muciniphila* improves gut barrier function associated with the stimulation of mucins, increase in thickness of the colonic mucus layer, and improvement of the enterocyte monolayer integrity [171,172]. Furthermore, it sustains intestinal barrier integrity, regulates host inflammatory responses caused by a high-fat diet, reduces low-grade inflammation in obese animal models and in patients with metabolic syndrome, and positively affects metabolic responses such as the production of beneficial SCFA [173,174,175,176,177,178]. Chang et al. [179] successfully co-encapsulated *Akkermansia muciniphila* 139 in succinate-grafted alginate doped with EGCG by spray-drying. *A. muciniphila* encapsulated in modified alginate with EGCG was significantly protected compared with free cells and the unmodified alginate-coated probiotics from sGIC for 90 min. It was shown that EGCG filled the pores and cracks in the microcapsules during the encapsulation process, and thus loss of viability caused by oxygen was blocked effectively due to the antioxidant capacity of EGCG. Further studies focused on co-encapsulation of polyphenols from green tea and other sources with lactic-acid-producing bacteria are listed in Table 1.

Resveratrol, curcumin, and quercetin belong to biologically important natural phenols, widely known for their antioxidant, anti-carcinogenic, anti-inflammatory, and cardio-protective properties. Apart from their individual benefits, probiotics together with natural phenols have been demonstrated to perform a synergistic effect on host digestive health, such as the recovery of GI tract homeostasis. Although curcumin has individual benefits, it suffers from poor bioavailability and rapid degradation because it is sensitive to environmental conditions [19,180,181]. Therefore, Su et al. [182] co-encapsulated *Lactobacillus rhamnosus* GG (ATCC 53103) and curcumin within a propylene glycol alginate-based hydrogel delivery system (PGA-β-lgNPs-Cur). PGA-β-lgNPs-Cur composite hydrogel helped to reduce the chemical degradation of curcumin and increased the survival of *L. rhamnosus* GG during UV light exposure and long-term storage. Over four weeks of storage, up to 91.3% of curcumin remained chemically stable and 9.7 log CFU/g cells survived. PGA-β-lgNPs-Cur composite was also able to impede the release of prebiotic curcumin in the first 60 min of exposure to sGIC activity. Up to 8.9 log CFU/mL of viable *L. rhamnosus* GG could be detected when trapped in the composite hydrogel matrix after incubation in sGIC for 180 min [182]. Resveratrol, a scavenger of reactive oxygen species (ROS) and other free radicals, is metabolized by hepatic and gut microbiota enzymes, the result of which can impact gut microbiota diversity and composition, including inhibiting *Enterococcus faecalis*, increasing the Bacteroidetes-to-Firmicutes ratio, and increasing the *Lactobacillus* and *Bifidobacterium* populations [183]. Vázquez-Maldonado et al. [184] co-encapsulated *Bacillus clausii* and resveratrol in inulin and lactose by spray-drying. Co-microencapsulation of *Bacillus clausii* with resveratrol showing good efficacy: 8.52 ± 0.10 log CFU/g for inulin and 8.62 ± 0.06 log CFU/g for lactose capsules. Resveratrol carried alone in inulin capsules showed the highest antioxidant activity (26%), and in co-encapsulated forms with bacteria showed similar activity against free radicals: 21% in inulin and 23% in lactose. However, the detrimental effects of quercetin co-encapsulated with probiotics on bacterial viability was observed by Chávarri et al. [185]. Cell viability and encapsulation yields were low after co-encapsulating *Bifidobacterium bifidum* and *Lactobacillus gasseri* with quercetin.

#### 3.2.3. Co-Encapsulation with Dietary Fibers

Soluble and insoluble dietary fibers are defined as non-digestible carbohydrate polymers of three or more monomeric units that resist digestion in the small intestine and are selectively utilized by host microorganisms in the large intestine, with beneficial effect on human health, including:

- non-starch polysaccharides: cellulose, hemicelluloses, pectins, hydrocolloids;
- resistant oligosaccharides: FOS, GOS, inulin (which can selectively promote the growth of *Bifidobacterium* spp. and *Lactobacillus* spp.), and other resistant oligosaccharides;
- resistant starch: consisting of physically enclosed starch, chemically and/or physically modified starches, retrograded amylose, and some types of raw starch granules;
- lignin associated with the DF polysaccharides;
- chemically synthesized fibers [17,189,190,191].

In everyday life, whole grains, fruits, nuts, pulses, and other kinds of vegetables represent the main food sources of dietary fibers. As powerful energy sources for most gut microbes, dietary fibers can directly alter species composition and colony size and prevent pathogen adhesion. In addition, the fermentation process can be altered by dietary fibers, which normally leads to production of key physiological metabolites such as SCFA (namely acetate, propionate, and butyrate). Thus, dietary fibers affect the supply of important metabolites and by-products for other microorganisms in a cross-feeding process. Healthy colonic microbiota is characterized by SCFA production, of which butyrate is utilized as the main energy source for colonocytes, stimulating their growth and also the production of cytokines, which maintain barrier integrity and function [83,192,193,194,195]. Increasing levels of SCFA in the gut helps to reduce the luminal pH, creating a desirable environment for beneficial bacteria, inhibiting the growth of pathogenic agents, and enhancing mineral absorption, vitamin bioavailability, and barrier function [63,192]. For example, luminal pH alteration can change the bacterial profile of acid-sensitive species and stimulate production of microbiota-derived butyrate by *Faecalibacterium prausnitzii* and *Eubacterium* as well as *Anaerostipes* and *Roseburia* species. Although, *Bifidobacterium* spp. are not able to produce butyrate, they are associated with a butyrogenic effect through cross-feeding between *Bifidobacterium* spp. and butyrate producing bacteria [61,196,197]. Emerging research is heavily focused on microbiota–gut–brain communication, the so-called “gut-brain axis”, which conceptually provides bidirectional signaling between the gut microbiota and the central nervous system (CNS) [17]. Dalile et al. [198] clearly reviewed the role of SCFA in microbiota-gut-brain cross-talk. Dietary fiber substrates for SCFA-producing bacteria are highlighted, and the effects of SCFA on signaling pathways, including neural, humoral, immune, and endocrine routes, are identified. For example, inulin and FOS are substrates for *Bacteroides* and *Faecalibacterium* fermenting species, whereas GOS are utilized by *Bifidobacterium*, and resistant starch by *Ruminococcus* and *Bacteroides*, etc.

In several studies, probiotics have been successfully incorporated together with different types of dietary fibers into microcapsules, enhancing their storage stability, protection during processing, and passage through the GI tract [199,200]. One of the most extensively studied dietary fibers is inulin, a thermally stable and poorly soluble form of fructan. Inulin confers protection from oxidative stress (e.g., indirectly scavenging ROS by enhancing SCFA production and preventing lipid peroxidation in the stomach). It has also been used as a building material for microcapsules in order to protect probiotic cargo. Therefore, inulin has a multifunctional character; in addition to serving as a coating material, it serves as a prebiotic substrate [201,202]. Xavier et al. [202] confirmed that 10% inulin is a suitable coating agent to protect microencapsulated *L. acidophilus* La-5 during the spray-drying process and sGIC. Atia et al. [128] studied the effect of inulin addition to alginate microcapsules and reported its ability to protect probiotic strains *Pediocuccus acidilactici* UL5, *L. reuteri,* and *L. salivarius*. Microcapsules with different inulin concentrations of 0%, 5%, 10%, 15%, and 20% (*w*/*v*) in 2% (*w*/*v*) alginate solution were prepared, and the most effective was the alginate matrix with 5% inulin. Antimicrobial and probiotic properties of bacterial strains were not affected by co-encapsulation, and protection against low pH was increased by the addition of inulin. Kumherová et al. [203] co-encapsulated *B. animalis* subsp. *lactis* BB-12 with inulin and/or ascorbic acid by an extrusion method in alginate or by emulsion in milk protein. Co-encapsulation in a protein matrix enriched with 1% (*w*/*w*) inulin and 0.5% (*w*/*w*) antioxidant ascorbic acid showed a higher survival rate of probiotic bacteria during sGIC when compared with free cells or bacteria encapsulated in alginate. Inulin was also successfully co-encapsulated with *Bifidobacterium* mixed cultures, *L. plantarum* CCTCC M 2,014,170 and *L. rhamnosus* GG, and bacterial survival and resistance to sGIC was enhanced [204,205,206]. Table 2 summarizes studies where inulin and other dietary fibers, including FOS, GOS, polydextrose, trehalose, hi-maize, rice bran, resistant starch, and lacticol, were studied to assess the protection of bacterial cargo and overall improved efficacy of synbiotic activity.

FOS and GOS from natural sources or enzymatically synthetized are popular compounds utilized by various food and medical industries because they are effective in combatting pathogens and are easily fermented by beneficial gut microbiota into SCFA [207]. Sathyabama et al. [208] co-encapsulated natural carbohydrate sources, namely sugar beet (rich in FOS) and chicory (rich in inulin and FOS), with probiotic strains *Enterococcus faecium* and *Staphylococcus succinus* in alginate by emulsification. This study reported that chicory beads were more stable while exposed to sGIC, but sugar beads resulted in a higher survival rate of probiotic strains under the action of bile. These are important considerations when designing microcapsules, since artificial food additives are also linked with the emergence of new epidemic pathogens, such as the trehalose microcapsule expansion of *C. difficile*.

## 4. Gut Microbiota Modulation by Immobilized Probiotics

**Figure 2:**
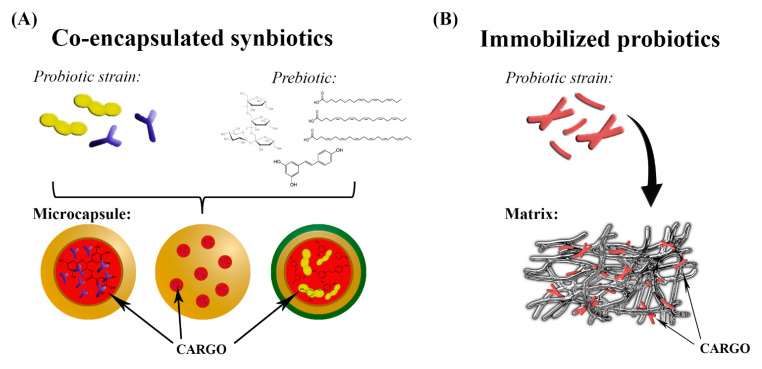
Schematic illustration of co-encapsulation (**A**) and immobilization (**B**) technologies.

Immobilization and encapsulation are two different processes, the terms of which are used interchangeably. Immobilization refers to the trapping of material within or throughout a carrier’s matrix; a small percentage of immobilized cargo is exposed to the environment at the carrier’s surface, and thus the immobilization process is not efficient in protecting the whole cargo. On the other hand, encapsulated cargo is contained within the coating material, which is formed continuously around an inner core matrix, as detailed in Section 3.2 (Figure 2). It is well established that the immobilization of probiotics enhances the viability of cultures and reduces the impact of environmental inactivating factors such as physicochemical changes during processing, storage, functional food production, and passage through the GI tract. Similarly to encapsulation, the biocompatible matrix used for immobilization should allow the bidirectional transport of nutrients and grow factors, as these are essential for cell metabolism and also for elimination of waste products [219,220,221,222]. The effectiveness of cell immobilization strategies depends mainly on the correct choice of the matrix used, which can be obtained from natural sources or manufactured. Various biocompatible supports have been used for the immobilization of lactic-acid-producing bacteria. Wheat grains, with their prebiotic character, provide the proteins, starch, dietary fibers, carbohydrates, minerals, and vitamins required for the development and preservation of bacteria and also promote human health. These were used as support for lactic-acid-producing bacteria by Sidira et al. [25,223,224] and Bosnea et al. [225]. Soybean grains, as a new type of support for the immobilization of *L. casei* CSL3, were used by Vitola et al. [226]. Milk proteins, such as whey protein and casein, can be used as natural carriers for microorganisms in functional food products, including yogurt or cheese, due to their structural and physicochemical properties. They were efficiently used as supports for immobilization of lactic-acid-producing bacteria [38,227,228] and kefir co-cultures [229,230]. However, the challenge in preparation of such dairy products is ensuring that sufficient numbers of viable probiotics are maintained until the product is consumed as well as during passage through the GI tract to its site of action [21,231]. Fruit pieces were previously also used as immobilization supports of lactic-acid-producing bacteria [221,222,232]. Fruit pieces contain natural prebiotic cellulose, which may contribute to cell survival and proliferation in the colon, thus enhancing the beneficial effects of the probiotics. Other types of fruit matrices are listed in Table 3. Jayani et al. [40] studied bacterial cellulose nanofiber as a delivery vehicle for the immobilization of *L. acidophilus* 016 through the adsorption-incubation technique. The viable cell count after 24 days of storage was 7.63 log CFU/g, compared with 10.72 log CFU/g immediately after immobilization. Bacterial cellulose exhibits exceptional properties, including high purity, high water retention capacity, a comprehensive crystalline network structure, good chemical stability, biocompatibility, and biodegradability, all of which are highly desired traits in many applications [40,233,234]. Furthermore, Nwagu et al. [235] used probiotic *Bacillus* sp. spores as immobilization support for bioactive agent bromelain. The immobilized bromelain showed significantly greater storage and thermal stability than the free bromelain. In follow-up research, Ugwuodo et al. [236] showed that the immobilized bromelain exhibited approximately 0.9-fold anti-inflammatory activity compared to free bromelain. Recent studies, summarized in Table 3, are focused on the viability of immobilized probiotics after exposure to sGIC and the potential use as a component of functional food products.

## 5. “Side Effects” of Gut Microbiota Functional Redundancy

It is also important to consider the contribution of stability, resistance, resilience, and redundancy features as it relates to the functional status of the native microbiota after any kind of intervention. If the gut microbiota is not resistant to disturbance, it alters its composition of species, genes, proteins, and functions. One way that microbiota is able to recover from such a functional disturbance is to promote growth and incorporate unrelated microbial species into the initial community; these unrelated taxa possess genes and proteins that are functionally redundant, promoting microbiota core functions despite compositional changes. For example, microbiota that are different at a compositional level show functional degeneracy by maintaining uniform profiles of proteins and metabolites [245,246,247,248]. Functional redundancy represents a natural ability of microbiota communities to restore core functions, emphasizing the need to closely monitor functional changes at the molecular level of host-microbe interactions. This becomes especially important when considering modulation of microbiota communities through microbial treatments that use co-microencapsulated synbiotics or immobilized probiotics.

## 6. Conclusions and Future Directions

Today, there is intense demand for the industrial production of multiple bioactive ingredients for co-encapsulation into microcapsules to enhance the stability and efficiency of probiotics as well as to decrease cost of the final product. Co-encapsulation of synbiotics (probiotic and prebiotic products) into single delivery systems has future profit-making potential, because numerous studies support their daily consumption to help to combat disease and maintain gut health and overall consumer well-being. However, several conditions need to be fulfilled in order to reduce losses during production of microcapsules and their subsequent application. First, encapsulation techniques and carrier materials need to be carefully selected. Suitable probiotic and prebiotic candidates with or without interdependency should be strategically chosen with precision microbial therapy in mind. The search for new prebiotic compounds and for the right combinations of prebiotics and probiotics with a synergistic effect on human health should be relentless, leaving no stone unturned. Despite the well-known extraordinary properties of synbiotics, additional in vivo and clinical trials are essential to demonstrate efficacy, and they need to be sufficiently powered with a randomized placebo control study design. To date, only few animal studies have been done to evaluate the effectiveness of co-encapsulated synbiotics in vivo [249,250,251]. This is particularly important when using microcapsules containing multiple bioactive ingredients, as the positive and negative interactions of synbiotics together with the encapsulation material become paramount to investigate. The mechanism of action of various co-encapsulated synbiotics in the host also need to be elucidated.

## Figures and Tables

**Table 1:** Studies of synbiotics comprising different types of phytochemicals.

Bioactive Substance	Probiotic Strain	Co-Encapsulation Technique	Carrier Material	Highlights	Ref.
GTE (rich in polyphenols)	*Lactobacillus helveticus* R0052	emulsification and internal gelation	whey protein and calcium pectinate	- with initial 0.5 mg/mL GTE concentration, 95.5% *L. helveticus* and 79% polyphenol EYs in MCs were observed - additional protection of *L. helveticus* during sGIC was significantly enhanced in MCs from pectin solutions coated with whey proteins and containing 1 mg/mL GTE	[137]
GTE (rich in polyphenols)	*Lactobacillus rhamnosus* GG	spray-drying	modified huauzontle’s starch and whey protein	- final count of the cells was 9.01 ± 0.03 log CFU/g within the MCs after spray-drying - with 0.1 mg/mL of ascorbic acid within MCs, 7.33 ± 0.16 CFU/mL of *L. rhamnosus* was maintained for five weeks of storage at 4 °C - 38.52 ± 0.72% of green tea polyphenols formed complex with at least one component of the MCs	[186]
Agar-based extract from Gelidium seaweed (rich in polyphenols)	*Bifidobacterium pseudocatenulatum* CECT 7765	emulsification and internal gelation	agar/agarose/whey protein/gelatin/starch	- the presence of polyphenols and proteins in the unpurified agar MCs significantly improved the *B. pseudocatenulatum* viability both at ambient and refrigerated storage conditions	[118]
Blackberry juice (rich in polyphenols and anthocyanins)	*Lactobacillus acidophilus* DSM13241	spray-drying	gum Arabic/maltodextrin/whey protein/50:50 blends	- 98.4 ± 1.0% total phenolic compounds and 99.0 ± 1.0% total monomeric anthocyanin content presented in gum Arabic and maltodextrin blend MCs - *L. acidophilus* survival was 81.2 ± 0.7% after ten weeks at 20 °C in whey protein MCs	[120]
Black currant extract (rich in anthocyanins, polyphenols, and flavonoids)	*Lactobacillus casei* ssp. *paracasei (L. casei* 431^®^)	freeze-drying	whey protein and inulin and chitosan	- 95.46% ± 1.30% EY for anthocyanins and 87.38% ± 0.48% EY for *L. casei* - viability after 90 days at 4 °C of the co-encapsulated cells with black currant extract ranged from 8.13 to 6.35 log CFU/g - anthocyanins were mostly released in the intestinal environment during sGIC	[136]
Apple skin extract (ASPE) (rich in polyphenols)	*Lactobacillus acidophilus*	co-extrusion	alginate	- EY for all the obtained alginate MCs was over 96%- the co-encapsulation of *L. acidophilus* with an aqueous or ethanolic ASPE protected cells in acidic conditions, with cell loss only 2.61 and 2.78 log CFU/g, respectively, in comparison with cell loss in MCs without ASPE (3.08 log CFU/g) and free cells (5.41 log CFU/g)	[187]
Cinnamon extract (PRCE) (rich in proanthocyanidin)	*Lactobacillus paracasei* (BGP1) *and Bifidobacterium animalis* subsp. *lactis* (BLC1)	complex coacervation followed by freeze drying	whey protein and gum Arabic	- the treatments with *B. animalis* and 5% PRCE presented greater EY for probiotic, phenolics, and proanthocyanids, with 98.59% ± 0.45, 119.49% ± 4.21, and 81.25% ± 1.9, respectively - higher viability of *B. animalis* (9.30 ± 0.16 log CFU/g) after 120 days of storage at 7 °C than *L. paracasei* (6.64 ± 0.10 log CFU/g)	[188]
Yellow onion skin extract (rich in flavonoids)	*Lactobacillus casei* ssp. *paracasei* (*L. casei* 431^®^)	freeze-drying	whey protein and inulin and maltodextrin	- EY of *L. casei* in MCs with flavonoids was 72.49 ± 0.11% - 85% of flavonoids in MCs were available after sGIC - stimulating effect on *L. casei* viability was observed after 21 days in soft cheese with MCs	[107]

Abbreviations used: ASPE, apple skin polyphenol extract; EY, encapsulation yield; GTE, green tea extract; MCs, microcapsules; PRCE, proanthocynidin cinnamon extract; sGIC, simulated gastrointestinal conditions.

**Table 2:** Probiotics co-encapsulated with dietary fibers.

Bioactive Substance	Probiotic Strain	Co-encapsulation Technique	Carrier Material	Highlights	Ref.
GOS (BiMuno^TM^)	*Bifidobacterium breve* NCIMB 8807	fluid-bed drying	alginate and chitosan and poly(D,L-lactic-co-glycolic acid)	- 6.6 ± 0.5 log CFU/mL cells of encapsulated *B. breve* survived 1 h in sGIC in alginate and chitosan MCs - 8.0 ± 0.3 log CFU/mL cells survived in MCs with GOS/poly(d,l-lactic-co-glycolic acid) included	[209]
Sugar beet	*Lactobacillus salivarius* NRRL B-30514	emulsification	sugar beet pectin	- 87% EY of *L. salivarius* in sugar beet pectin MCs prepared in sugar beet pectin/soybean oil/water emulsions - after 2 h incubation in sGIC, the lowest decrease in viability was observed in emulsion with CaCl_2_ - free *L. salivarius* became undetectable after 3 h in sGIC - cross-linking sugar beet pectin by Ca^2+^ ions additionally protected *L. salivarius* during sGIC	[117]
Lactitol, GOS, eight types of commercial prebiotics	*Lactobacillus casei* 28-2, *Lactobacillus casei* 30-1, *Lactobacillus paracasei* 6062, *Lactobacillus plantarum* 25-1	extrusion	alginate and chitosan	- lacticol had a highest prebiotic score value for *Lactobacillus* strains - mechanical strength of MCs with different lacticol additions decreased constantly in sGIC - log reduction of cells after 120 min in sGIC was 7.37, 3.41, 3.13, and 2.97 for 0, 10, 20, and 25 g/L concentration of lacticol in MCs, respectively	[210]
Inulin, polydextrose	*Lactobacillus acidophilus* 04	spray-chilling	lipid matrix	-free cells were not detectable after 210 min in sGIC -ca. 60% of the cells in the MCs with or without a prebiotic were viable after 300 min in sGIC	[122]
Inulin, GOS	*Lactobacillus acidophilus* 5 and *Lactobacillus casei* 01	extrusion	alginate and chitosan	-the presence of 1.5% GOS in the MCs provided the best protection with only 3.1 and 2.9 log CFU/g reduction for *L. acidophilus* 5 and *L. Casei* 01, respectively, after incubation in sGIC	[211]
Inulin, polydextrose	*Bifidobacterium* BB-12	spray-drying	sweet whey protein	-after sGIC, the free cell count showed a decrease of 1.18 log CFU/g, while the MCs showed decreases of 0.49, 0.97, and 2.45 log CFU/g for sweet whey, sweet whey and inulin, and sweet whey and polydextrose, respectively	[212]
Inulin	*Lactobacillus casei* 431	extrusion	alginate and chitosan	- 5.7 log reduction for free cells, 3.9 log reduction for alginate MCs, 2.7–2.8 log reduction for alginate and inulin MCs, 0.7–0.9 log CFU/g reduction for alginate and inulin MCs coated with chitosan after exposition to sGIC	[213]
Inulin, hi-maize, trehalose	*Lactobacillus acidophilus* La-5	spray-drying	gum Arabic and maltodextrin and inulin/hi-maize/trehalose	- MCs produced with hi-maize showed the greatest viability after sGIC, from 11.50 ± 0.09 to 10.49 ± 0.12 log CFU/g, followed by inulin, from 11.38 ± 0.11 to 10.16 ± 0.08 log CFU/g	[214]
Inulin	*Lactococcus lactis subsp. lactis* R7	spray-drying	whey protein and inulin	- 94.61% EY of *L. lactis* in MCs - free cells exposed for 7 days to pH 2.0, 2.5, and 3.0 had 2.18, 1.00, and 1.78 log CFU/g reduction, respectively; in contrast, no significant decrease of co-encapsulated *L. lactis* was observed	[215]
Inulin, resistant starch	*Lactobacillus plantarum* ATCC 8014™ and *Bifidobacterium animalis* subsp. *lactis*	electro-hydrodynamic atomization	alginate and chitosan	- MCs containing resistant starch were better in maintaining the viability of probiotics under sGIC - viability of *B. lactis* in MCs with resistant starch was reduced from 8.77 ± 0.12 to only 7.19 ± 0.15 CFU/g	[127]
Inulin, hi-maize, rice bran	*Lactobacillus acidophilus* LA-5	extrusion/external ionic gelation	alginate or blends with (rice bran/inulin/hi-maize)	- initial count of *L. acidophilus* was 13.85 ± 0.05, 13.94 ± 0.20, 14.24 ± 0.05, and 11.21 ± 0.09 log CFU/g for alginate, rice bran, inulin, and hi-maize, respectively, and after exposure to sGIC: 11.18 ± 0.13, 8.06 ± 0.01, 8.93 ± 0.09, and 9.47 ± 0.23 log CFU/g, respectively - the alginate, rice bran, and hi-maize MCs maintained viable probiotics for 120 days at 25 °C; rice bran and inulin preserved viable probiotics in MCs over the 120 days of storage at 7 °C; only in MCs with inulin did cells remain viable for 120 days at −18 °C	[42]
Inulin, hi-maize, rice bran	*Lactobacillus acidophilus* LA-5	emulsification/internal ionic gelation	pectin	- the best EY was obtained in MCs with rice bran and inulin: 91.24% and 90.59%, respectively - 3.30 log reduction in viability of free cells after the sGIC; however, in co-encapsulated *L. acidophilus,* only 0.11, 0.9, 1.63, and 2.37 log CFU/g reductions were observed for the pectin MCs or in formations with hi-maize, inulin, and rice bran, respectively	[216]
Inulin, hi-maize, rice bran	*Lactobacillus acidophilus* LA-5	emulsification/internal ionic gelation followed by freeze-drying	pectin	- the highest EY was obtained in MCs with inulin: 68.1%; 3.4 ± 0.1 log reduction in viability of free cells after sGIC and for co-encapsulated ones: 1.3 ± 0.2, 0.1 ± 0.0, 1.6 ± 0.2, and 1.0 ± 0.2 log CFU/g for pectin MCs or in formations with hi-maize, inulin, and rice bran, respectively, in relation to initial counts	[217]
Inulin	*Lactobacillus rhamnosus* ATCC 7469	freeze-drying	whey protein and crystalline nanocellulose and inulin	- the highest EY was 89.60% for formulation: whey protein—57.22%, crystalline nanocellulose—25.00%, and inulin—17.78%; this composition significantly improved survival of the probiotics in the sGIC in comparison with free cells	[218]

Abbreviations used: EY, encapsulation yield; GOS, galacto-oligosacharides; MCs, microcapsules; sGIC, simulated gastrointestinal conditions.

**Table 3:** Recent reports about viability of immobilized probiotics in simulated gastrointestinal conditions.

Carrier Material	Probiotic Strain	Simulated Gastrointestinal Conditions	Ref.
Apple pieces	*Lactobacillus casei* ATCC 393	- counts of immobilized *L. casei* were significantly higher after 120 min at pH 2.0 and after 30, 60, 90, and 120 min at pH 1.5 compared to free cells; cell immobilization resulted in significantly higher survival rates in pancreatic juices supplemented with 0.45% bile salts after 240 min and in bile salts after 120 min; reduced counts of staphylococci, enterobacteria, coliforms, and streptococci in rat feces after oral administration of free or immobilized *L. casei* contained in probiotic-fermented milk revealed modulation of gut microbiota	[54]
Apple disks	*Lactobacillus salivarius* spp. *salivarius* CECT 4063	- dried apple with immobilized encapsulated *L. salivarius* was mainly affected by the acidic environment created (10 mL of pepsin (0.6% *w*/*v*) adjusted to pH 3 with HCl 4 M) and the addition of bile; survival of immobilized *L. salivarius* also decreased with storage time at different gastro-intestinal stages	[237]
Dehydrated fruits: pineapple, guava, and kiwi	*Lactobacillus casei* CSL3	- the most appropriate support for immobilization of *L. casei* was pineapple, depending on viability and sensorial evaluation; sGIC did not affect viability of probiotics incorporated in cheese, either in its free or immobilized form	[238]
Sea buckthorn berries (Hippophae rhamnoides L.)	*Lactobacillus casei* ATCC 393	- immobilized *L. casei* remained at concentration 7.47 log CFU/g, while the free cells remained at 6.01 ± 0.13 CFU/g after sGIC - 90 days of frozen storage did not affect viability of *L. casei* incorporated in frozen yogurt, either in its free or immobilized form	[239]
Poly-γ-glutamic acid (γ-PGA)	*Bifidobacterium longum* NCIMB 8809 and *Bifidobacterium breve* NCIMB 8807	- both strains, protected with 2.5% γ-PGA, survived in simulated gastric juice (pH 2.0) with a slight reduction (<0.47 log CFU/mL) or no significant reduction after 4 h, while free cells died within 2 h - loss in viable cells of γ-PGA-immobilized *B. breve* and *B. longum* showed only around 0.5 log and 1.1 log CFU/mL reductions, respectively; however, around 4.0 log and 3.4 log CFU/mL reductions were observed in free *B. breve* and *B. longum* cells, respectively, after 13 days of storage in orange juice at 4 °C	[240]
Bacterial cellulose (BC) (produced by Gluconacetobacter xylinus)	*Lactobacillus delbrueckii* PKM 490, *Lactobacillus plantarum* DSM 13,273, and *Lactobacillus casei* ATCC 393	- the immobilization of *Lactobacillus* in BC during co-culture with cellulose-synthetizing *G. xylinus* enabled almost full protection of the probiotic bacteria against the harmful environment of sGIC - co-cultures of *G. xylinus* and *Lactobacillus* strains did not adversely influence the BC biosynthesis	[241]
White, milk, and dark chocolate	*Lactobacillus casei* 01 and *Lactobacillus acidophilus* LA-5	- the immobilized *L. casei* in different chocolates had higher levels of survivability after being exposed to sGIC, and they still remained to be viable at ~2 log CFU/mL after 6 h - the survival rate of *L. casei* was generally higher than that of *L. acidophilus*, regardless of the types of chocolate; however, storage conditions at 4 °C were suitable for retaining probiotic viability, no matter the probiotic strain or chocolate type used	[242]
Wheat bran	*Lactobacillus casei* ATCC 393	- incubation for 2 h in the simulated gastric acid led to higher reduction of viability of free cells than immobilized ones - similarly, enhanced viability of immobilized *L. casei* incorporated into the yogurt samples during simulated gastric juice conditions (from 9.94 log CFU/g to 9.27 log CFU/g) compared to the free cells (from 9.50 log CFU/g to 8.22 log CFU/g) was observed	[243]
Wheat bran	*Lactobacillus casei* ATCC 393	- incubation for 2 h in simulated gastric juice (pH 3) of cheese samples with the freeze-dried immobilized *L. casei* resulted in a low loss of cell viability (from 8.43 to 8.19 log CFU/g), while in the case of cheese containing free *L. casei*, the loss of cell viability was higher (from 8.24 to 7.82 log CFU/g)	[22]
MCPPM, MCP, MC	*Lactobacillus plantarum* NCIMB 8826	- at an adjusted simulated gastric fluid (pH 3.0), reduction in the viability of free cells was 4.4 log CFU/g after 180 min, while the immobilized *L. plantarum* had reductions of 1.0, 1.1, and 1.6 log CFU/g for MCPPM, MCP, and MC, respectively - after 24 h exposure to 1% bile juice, 4.2, 1.9, 1.7, and 1.8 log CFU/g reductions were observed for free cells, MC, MCP, and MCPPM, respectively	[244]

Abbreviations used: BC, bacterial cellulose; MCs, microcapsules; MC, maize:cowpea; MCP, maize:cowpea:peanut; MCPPM, maize:cowpea:peanut:powdered milk; sGIC, simulated gastrointestinal conditions; γ-PGA, poly-γ-glutamic acid.

## Data Availability

Data sharing not applicable.
